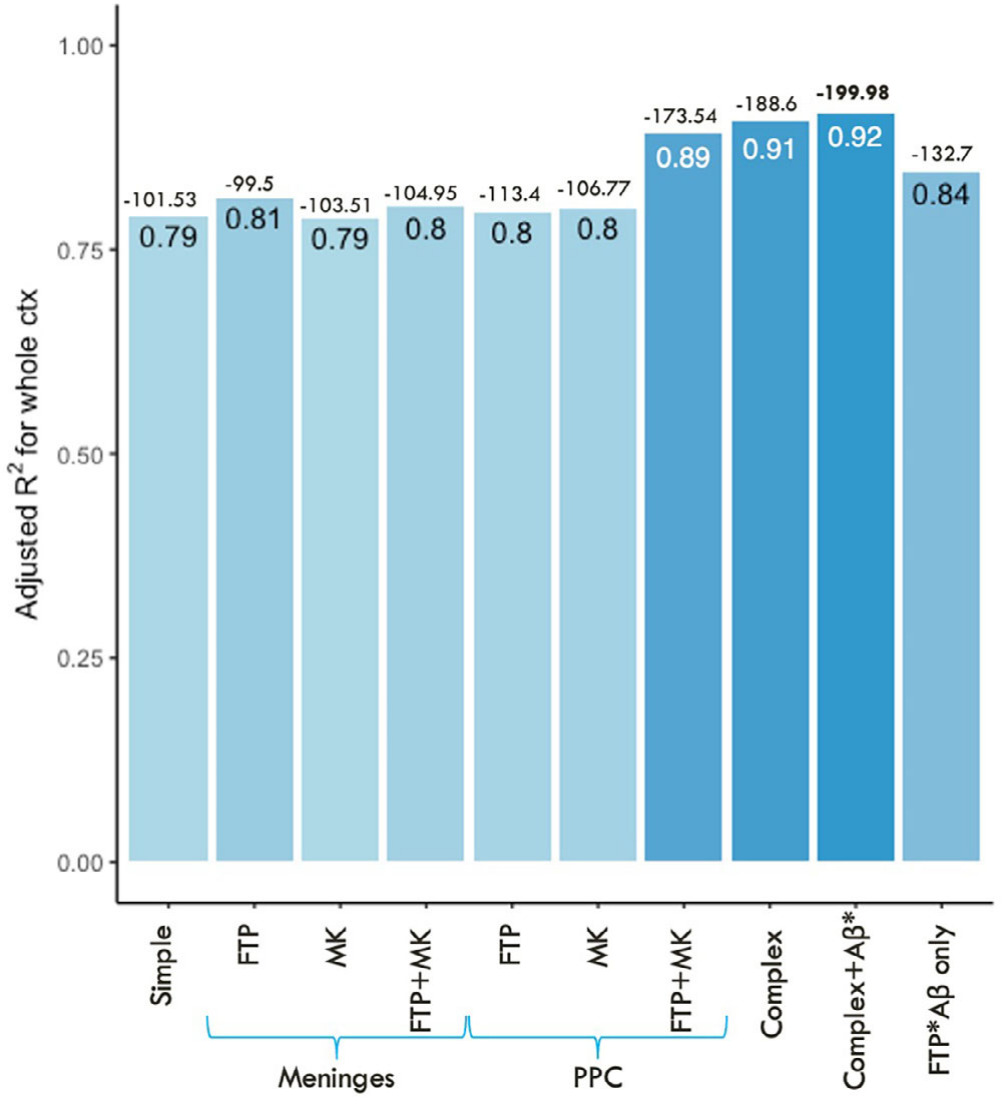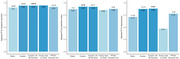# Biological Factors Influencing [18F]MK6240 and [18F]FTP Correlation in Target Regions

**DOI:** 10.1002/alz.087641

**Published:** 2025-01-09

**Authors:** Cécile Tissot, Hsin‐Yeh Tsai, Joseph Therriault, Dana Tudorascu, Nesrine Rahmouni, Stijn Servaes, Jenna Stevenson, Firoza Z Lussier, Stefania Pezzoli, Jacob Ziontz, Brian A. Gordon, Belen Pascual, Val J. Lowe, David N. soleimani‐meigooni, Hwamee Oh, William E Klunk, Pedro Rosa‐Neto, William J. Jagust, Tharick Ali Pascoal, Suzanne L. Baker

**Affiliations:** ^1^ Lawrence Berkeley National Laboratory, Berkeley, CA USA; ^2^ Lawrence Berkeley National Lab, Berkeley, CA USA; ^3^ McGill University, Montreal, QC Canada; ^4^ University of Pittsburgh, Pittsburgh, PA USA; ^5^ University of California, Berkeley, Berkeley, CA USA; ^6^ Washington University in St. Louis School of Medicine, St. Louis, MO USA; ^7^ Houston Methodist Research Institute, Houston, TX USA; ^8^ Mayo Clinic, Rochester, MN USA; ^9^ Memory and Aging Center, Weill Institute for Neurosciences, University of California, San Francisco, San Francisco, CA USA; ^10^ Brown University, Providence, RI USA

## Abstract

**Background:**

The association between [^18^F]Flortaucipir (FTP) and [^18^F]MK6240, two commonly used tau‐PET tracers in Alzheimer’s disease (AD), varies due to distinct binding properties and off‐target signal regions. Our study aims to elucidate the biological factors influencing this association and evaluate the applicability of a common equation across different on‐target regions.

**Method:**

113 individuals from the HEAD dataset (11 young, 58 cognitively unimpaired elderly, and 44 cognitively impaired) underwent [^18^F]MK6240, [^18^F]FTP and Aβ‐PET scans. Images were processed using the inferior cerebellar grey (CG) as the reference region, with subjects categorized by Aβ status. Stepwise linear fitting identified off‐target (OFF) regions impacting tracer associations within the whole cortex, Braak and temporal‐meta regions of interest (ROIs). OFF regions included were a composite mask (pallidum, putamen, caudate ‐ PPC) to address collinearity in subcortical nuclei, as well as the region with the greatest partial volume effects (PVE) on the ROI (meninges, choroid plexus, and the hotspot medial to entorhinal cortices (EC)). Akaike information criteria (AIC) analyses tested linear models with various off‐target regions.

**Result:**

PPC and PVE regions influenced tracer association in on‐target regions. PPC binding is associated with age. PPC and meninges retention, along with Aβ status interaction, demonstrated the best model fit for the whole cortex (Figure 1). Weighting factors derived from the whole cortex model were applied to Braak regions and temporal meta‐ROI. Braak III‐VI and temporal‐meta ROI showed optimal models including OFF regions and Aβ status interaction (Figure 2). Forcing the whole equation gave optimal results. For Braak I (EC), adding the OFF covariates only was the best model. For Braak II (hippocampus), the best model included OFF regions and the interaction with Aβ status (Figure 2). Forcing the whole cortex equation onto Braak I and Braak II yielded suboptimal fits.

**Conclusion:**

Harmonizing tau‐PET tracers involves considering significant covariates. Integrating meningeal and age‐related off‐target regions enhances [^18^F]MK6240 and [^18^F]FTP association in on‐target regions, further improved by Aβ status inclusion. While the whole cortex model may not suit early ROI, it proves effective for Braak III and beyond. Creating an equation for tracer translation necessitates incorporating meningeal and subnuclei off‐target retention.